# Functionality and the evolution of marginal stability in proteins: Inferences from lattice simulations

**Published:** 2007-01-16

**Authors:** Paul D. Williams, David D. Pollock, Richard A. Goldstein

**Affiliations:** 1 Department of Chemistry, University of Michigan, Ann Arbor, MI, 48109, USA;; 2 Department of Biological Sciences, Louisiana State University, Baton Rouge, LA, 70803, USA;; 3 Mathematical Biology, National Institute for Medical Sciences, The Ridgeway, Mill Hill, London MW7 1AA, UK

**Keywords:** lattice models, protein thermodynamics, molecular evolution

## Abstract

It has been known for some time that many proteins are marginally stable. This has inspired several explanations. Having noted that the functionality of many enzymes is correlated with subunit motion, flexibility, or general disorder, some have suggested that marginally stable proteins should have an evolutionary advantage over proteins of differing stability. Others have suggested that stability and functionality are contradictory qualities, and that selection for both criteria results in marginally stable proteins, optimised to satisfy the competing design pressures. While these explanations are plausible, recent research simulating the evolution of model proteins has shown that selection for stability, ignoring any aspects of functionality, can result in marginally stable proteins because of the underlying makeup of protein sequence-space. We extend this research by simulating the evolution of proteins, using a computational protein model that equates functionality with binding and catalysis. In the model, marginal stability is not required for ligand-binding functionality and we observe no competing design pressures. The resulting proteins are marginally stable, again demonstrating that neutral evolution is sufficient for explaining marginal stability in observed proteins.

## Introduction

It has been repeatedly observed that a high proportion of globular proteins are marginally stable under physiological conditions, with a Δ*G*_folding_ of about −5 to −10 kcal/mol. (Brandts 1967; Privalov and Khechinashvili 1974; Savage et al. 1993; Ruvinov et al. 1997; Vogl et al. 1997; Giver et al. 1998). This is in spite of several factors that suggest that stable proteins might have advantages over marginally stable proteins. For instance, flexible proteins may be less resistant to proteolysis (Fontana, Polverino de Laureto, and De Filippis 1993; Hubbard, Eisenmenger, and Thornton 1994; Fontana et al. 1997; Hubbard, Beynon, and Thornton 1998), denaturation (Wagner and Wuthrich 1979), detrimental conformational change (Carrell and Lomas 1997; Dobson 2001; Bucciantini et al. 2002), aggregation (Lomas and Carrell 2002), and loss of active-site integrity. In addition, binding between less stable and more flexible proteins and their corresponding ligands requires strong binding interactions. More stable proteins do not need such strong binding energies because they lose less entropy upon binding (Schulz 1979). These consequences of higher stability might be expected to increase the evolutionary success of organisms containing stabilised forms of these proteins, suggesting that highly stable proteins should be more common.

The fact that most proteins are not highly stable suggests that other factors are involved, and several hypotheses have been developed to explain this discrepancy. Most of these hypotheses centre on various reasons why marginally stable proteins would have a selective advantage over more stable proteins. For instance, it has been suggested that proteins have evolved towards marginal stability in order to function, suggesting that there is a narrow range of stability consistent with functionality (Rasmussen et al. 1992; Tsou 1998; Zavodszky et al. 1998). There are several reasons why functionality might be limited to proteins of marginal stability. Marginally stable proteins might be more flexible (Wagner and Wuthrich 1979; Tang and Dill 1998), increasing functionality by enabling the formation of strong binding interactions with specific ligands or by providing flexibility needed for conformational change (Lipscomb 1970; Artymiuk et al. 1979; Frauenfelder, Petsko, and Tsernoglou 1979; Wrba et al. 1990; Varley and Pain 1991; Daniel, Dines, and Petach 1996; Zavodszky et al. 1998; Daniel and Cowan 2000). It has also been suggested that marginal stability may be advantageous because ligand binding with marginally stable proteins is comparatively difficult. Binding affinities and specificities of less stable proteins might be more readily adjusted by mutation, phosphorylation or other processes, or selectivity might be enhanced by allowing binding only in the presence of specific interactions (Dunker et al. 1998; Wright and Dyson 1999; Dunker and Obradovic 2001; Dunker and others 2001; Namba 2001). In addition, the physiological importance of marginal stability might involve considerations other than maximizing functionality or selectivity. For instance, unstable proteins might provide more rapid turnover than stable proteins.

A second class of explanations involves the hypothesis that marginal stability is the result of a trade-off between functionality and stability, that the constraints on the amino acids imposed by functionality reduce the opportunities to produce a highly-stable protein resulting in a negative correlation between functionality and stability. This could result if functionality required specific amino acids at functionally-important locations that were incompatible with high stability, so that large numbers of possible sequences, including those with high stability, would be excluded from the evolutionary dynamics. It has been observed, for instance, that mutations increasing protein stability and activity are much more rare than mutations increasing either separately (Alber and Wozniak 1985; Bryan et al. 1986; Liao, McKenzie, and Hageman 1986; Shoichet et al. 1995), although the presence of mutations that increase both (Giver et al. 1998) indicates that functionality and stability are not mutually exclusive. If this trade-off holds for all protein sequences, marginal stability is prevalent because it provides the best balance between the sequences that result in stability and functionality.

These explanations generally arise from an ‘adaptationist’ paradigm, which is to say that the observation of marginal stability in proteins requires an explanation of how this contributes to the reproductive fitness of the organism, either as a direct adaptation or as optimization given constraints. Random events and processes, however, are important factors in the dynamics of evolution and can influence the characters that result (Sueoka 1962; Kimura 1968; King and Jukes 1969). Gould and Lewontin have emphasised the importance of examining possible alternatives to adaptationist selection (Gould and Lewontin 1979). Specifically, they stress that the present usefulness of a character may belie its origins, so that one should avoid ascribing characters to adaptation simply due to their present use. Random events may have led to the existence of the character, which was used to advantage only later. Before adaptation can be judged the cause of the emergence of a character, other explanations must be ruled out.

Consistent with these ideas, a third explanation of the observed marginal stability in proteins involves the concept of regions of sequence space and what has been termed ‘designability’ (Govindarajan and Goldstein 1995; Govindarajan and Goldstein 1996; Li et al. 1996; Buchler and Goldstein 1998; Shakhnovich 1998; England, Shakhnovich, and Shakhnovich 2003). The idea is that the ‘sequence entropy’ or volume of sequence space corresponding to any property influences whether this property is likely to result from evolutionary dynamics. This idea has been applied to the distribution of different structures (Finkelstein and Ptitsyn 1987; Govindarajan and Goldstein 1995; Govindarajan and Goldstein 1996; Li et al. 1996), the question of whether proteins fulfill the thermodynamic hypothesis (Govindarajan and Goldstein 1998), the stability of proteins, (Taverna and Goldstein 2002), ligand binding properties (Blackburne and Hirst 2001; Williams, Pollock, and Goldstein 2001; Blackburne and Hirst 2003; Bloom et al. 2004), and the ability of proteins to explore a range of different structures (Govindarajan and Goldstein 1997a; Govindarajan and Goldstein 1997b; Bornberg-Bauer and Chan 1999; Taverna and Goldstein 2000; Deeds, Dokholyan, and Shakhnovich 2003; Tiana et al. 2004; Shakhnovich et al. 2005). In previous work exploring the evolution of lattice proteins with a fitness function dependent only on stability, it was found that sequence entropy was a sufficient explanation for the observation of marginal stability in proteins, and that this effect would favor mechanisms of function consistent with marginal stability (Taverna and Goldstein 2002). In further work, we developed a simple model with fitness represented by the ability of the protein to bind a ligand, observing similar results (Williams, Pollock, and Goldstein 2001).

To examine these hypotheses in the context of protein functionality and to extend the previous work, we simulate the evolution of proteins using a lattice-protein model of protein-folding and ligand-binding that allows the study of protein stability and function. In these simulations, the fitness function is based on diffusion-limited reaction kinetics. The model is designed so that fitness increases with binding strength, which tends to increase with stability, meaning that marginally stable proteins have no evolutionary advantage over proteins of greater stability. We also observe no design constraints or trade-offs between stability and functionality among these evolved proteins. Our evolutionary simulations, however, lead to marginally stable proteins. This indicates again, with a more realistic simulation, that random evolution is sufficient to generate marginal stability. This does not prove that marginal stability is not an adaptation, but rather demonstrates that marginal stability could result in the absence of any adaptive role, that its presence does not indicate that it plays such an adaptive role, and therefore that marginal stability is not a phenomenon that needs an explanation based on evolutionary advantage.

## Methods

### Protein Model

Protein models should accurately represent important and relevant aspects of real proteins yet be simple enough for rapid computational evaluation. Our model must be relatively simple indeed, as evolution simulations involve the examination of many protein sequences over a large number of generations. To examine the previously described hypotheses, the model must map protein sequence to stability in a compact state as well as the propensity for binding and acting upon a specified ligand.

The details of this model have been more thoroughly described elsewhere (Williams, Pollock, and Goldstein 2001). Each model protein consists of a chain of 16 amino acids on a 2-dimensional square lattice. While the 2-dimensional model is problematic for dynamics simulations (Shakhnovich 1997), for thermodynamic analyses involving sums over states it is more accurate at representing the appropriate number of buried vs. exposed residues. Intra-protein contacts are defined as non-sequentially-adjacent residues one lattice-unit apart in distance. Compact structures have nine contacts (the maximum number possible for a 16-residue protein) and fit in a square with four residues per side. All 802,075 possible structures are considered, of which only 69 are compact.

The free energy *G*(*k*) of a sequence {*A*_1_, *A*_2_…*A*_16_} in conformation *k* is given by

(1) $$G(k)=\sum_{r<s}\gamma (A_{r},A_{s})Q_{r,s}^{k}$$

where γ (*A_r_*, *A_s_*) is the contact potential between amino acids *A_r_* and *A_s_*, and where *Q_r_*_,_*_s_^k^*, is 1 if residues *r* and *s* are in contact in structure *k*, and is otherwise 0. The contact potentials are obtained from the statistical analysis of Miyazawa and Jernigan, who developed a contact-potential matrix that describes the interactions between amino acids (Miyazawa and Jernigan 1985). Due to the nature of this statistical analysis, these potentials represent ‘potentials of mean force’, implicitly including hydrophobic interactions and other effects of the solvent. They therefore represent contributions to the free energy rather than enthalpy. In this matrix, the influence of covalent cysteine crosslinks is shown by the high magnitude of the Cys-Cys potential. As such binary interactions are incompatible with the contact potential as encoded in our model, and would significantly change the number and character of allowed conformations, we do not consider them in our model. To account for this, we use a modified potential matrix where the Cys-Cys potential has been replaced by the Ser-Ser potential. In addition, the values in the matrix have all been multiplied by two to counteract the effect of the limited number of two dimensions.

We use Boltzmann statistics to determine *P*(*k*), the thermodynamic probability of folding into conformation *k*, assuming all conformations are in equilibrium:

(2) $$P(k)=\frac{\text{exp}\left(\frac{-G(k)}{k_{B}T}\right)}{\sum_{|\text{All onformation }k^{\prime }}\text{exp}\left(\frac{-G(k^{\prime })}{k_{B}T}\right)}.$$

where *T* is the temperature and *k_B_* is Boltzmann’s constant, and again the sum in the denominator is over all structures, both compact and extended. *P*(Compact) is defined as the sum of probabilities of all compact structures; the change in free energy upon folding into a compact state is then

(3) $$\Delta G(\text{Compact})=-k_{B}T\text{ln}\left(\frac{P(\text{Compact})}{1-P(\text{Compact})}\right)$$

We assume that the conformation with the lowest free energy should be the native state of the protein; since we are mainly interested in compact structures, the compact structure with the lowest free energy shall be referred to as the native state (Govindarajan and Goldstein 1998).

**Figure 1:**
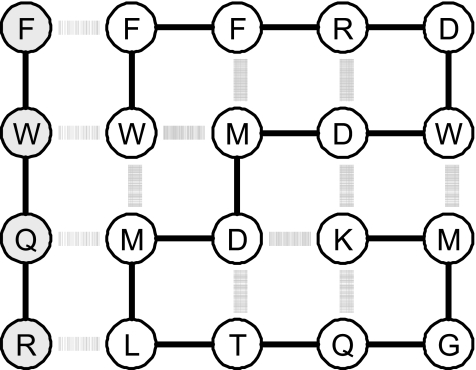
An example of a model protein in a compact conformation bound to a ligand, shown in grey. Covalent bonds are shown as solid lines, contact interactions as thick (intramolecular) and thin (intermolecular) stripes.

We model protein-ligand binding as a four-residue peptide contacting any of the four sides of a compact protein, such that maximal contact between the ligand and the face of the protein is made, as illustrated in Figure 1. The ligand may face either of two directions on any of the four sides of a conformation, so there are 69 × 4 × 2 = 552 possible binding sites on a protein sequence. The free energy of a complex where the protein is in compact conformation *k* and the ligand is bound to site *l* is

(4) $$G(k,l)=G(k)+\sum_{r}^{16}\sum_{q}^{4}\gamma (A_{r},A_{q})Q_{r,q}^{k,l}$$

where *q* is over the four locations in the peptide ligand, and *Q^k,l^_r,q_* is equal to 1 if residue *r* in the protein is in contact with residue *q* in the ligand in this particular bound conformation. We use Boltzmann statistics to determine the probability that the protein binds the ligand

(5) $$\begin{array}{l} {\text{P}}^{0}(\text{Bound})=\\ \frac{\sum_{k,l}\text{exp}\left(\frac{-G(k,l)}{k_{B}T}+\frac{\Delta S_{\text{lig}}}{k_{B}}\right)}{\sum_{k^{4}}\text{exp}\left(\frac{-G(k^{\prime })}{k_{B}T}\right)+\sum_{k,l}\text{exp}\left(\frac{-G(k,l)}{k_{B}T}+\frac{\Delta S_{\text{lig}}}{k_{B}}\right)} \end{array}$$

where Δ*S*_lig_ is the concentration-dependent change in the entropy of the ligand upon binding. We represent the probability of ligand bound by *P*^0^(Bound) to indicate that this is calculated without considering any forward reactions, that this assumes an equilibrium between the bound and unbound forms.

Under conditions when very little of the protein is bound to ligand, we can ignore the second term in the denominator and calculate the relative probability of the protein binding the ligand for a fixed concentration by multiplying *P*^0^(Bound) by exp(−Δ*S*_lig_/*k_B_*), yielding

(6) $$\begin{array}{l} P_{\text{rel}}^{0}(\text{Bound})\equiv \text{exp}\left(-\frac{\Delta S_{\text{lig}}}{k_{B}}\right)P^{0}(\text{Bound})\\ \approx \frac{\sum_{k,l}\text{exp}\left(\frac{-G(k,l)}{k_{B}T}\right)}{\sum_{k^{\prime }}\text{exp}\left(\frac{-G(k^{\prime })}{k_{B}T}\right)}. \end{array}$$

### Evolution Model

We model a population of random proteins evolving through mutation and replication. Starting with an initial population of 1000 protein sequences, we allow a fixed rate of mutations, modeled as a Poisson distribution with a mean of 20 mutations per generation. Sequences are then replicated according to their fitness, as described below. The population size is maintained at a constant level of 1000 proteins throughout the experiment.

### Measure of Fitness

Many factors affect the evolutionary success of a protein, ranging from the intrinsic properties of the protein to external and indirectly related circumstances. For this paper, we are concerned only with the effects of selection for protein functionality. To study these effects, we construct a fitness function based on the rate of catalysis of bound ligands.

Assuming that product-formation is beneficial, we consider fitness directly proportional to the rate of catalysis. We model this rate with Michaelis--Menten kinetics, corresponding to reactions of the following type,

(7) $$P+L\overset{k_{D}}{\underset{k_{\text{uni}}}{⇄}}PL\overset{k_{2}}{\to }P+\text{Product}$$

where *P*, *L*, and *PL* are the protein, ligand, and protein-ligand encounter complex, respectively, and *k_D_*, *k*_uni_, and *k*_2_ are the rates of diffusional encounter, unimolecular dissociation, and catalysis, respectively. While our protein-ligand binding model is designed for the calculation of thermodynamic probabilities and cannot be used to explicitly calculate the kinetics of folding or binding processes, we assume that *k_D_* does not depend upon the strength of binding, so that as *P*^0^(Bound) increases, *k*_uni_ should decrease to satisfy the conditions for equilibrium.

We are interested in investigating the ease with which marginal stability could result in evolving proteins in the absence of any specific advantage to marginal stability. For this reason, we assume that *k*_2_ is independent of binding strength. In this case, the rate of catalysis, and thus the fitness used in the evolution simulations, is given by

(8) $$\text{Fitness}=\frac{1}{1+\frac{P^{0}{\left(\text{Bound}\right)}_{1/2}}{P^{0}(\text{Bound})}}=\frac{1}{1+\frac{P_{\text{rel}}^{0}{\left(\text{Bound}\right)}_{1/2}}{P_{\text{rel}}^{0}(\text{Bound})}}$$

where *P*^0^(Bound)_½_ is equal to

(9) $$P^{0}{\left(\text{Bound}\right)}_{1/2}=\frac{k_{D}[L]}{k_{2}}$$

and *P*^0^_rel_(Bound)_½_ ≡ exp(−Δ*S*_lig_)*P*^0^ (Bound)_½_. (See Appendix for a detailed derivation.)

Fitness increases monotonically with increasing *P*^0^_rel_ (Bound), and approaches the maximum value asymptotically. This asymptotic domain represents the diffusion-limited nature of Michaelis-Menten kinetics - at a certain point, better ligand-binding will not result in faster catalysis.

In our evolution runs, proteins are selected for their ability to bind a specific ligand. The sixteen four-residue permutations of glutamate and lysine can form the strongest binding interactions of any ligand, while the optimal binding interaction of a polyalanine ligand is the weakest possible. We performed evolution runs using the ligands AAAA, EEEE, and EKEK, to examine the influence ligand-choice has on evolution. Real proteins have a wide range of *k*_2_ values, and act on ligands of varying concentration, diffusion rates, and Δ*S*_lig_. To account for this variety, we performed simulations with a range of values for *P*^0^_rel_ (Bound)_½_. For AAAA, *P*^0^_rel_(Bound)_½_ was 1.25, 12.5, 50, and 100, for EEEE it was 1.25, 100, 400, 3750, 7500 and 15,000, and for EKEK it was 1.25, 100, 400, and 3750.

In addition to performing evolution experiments, we also optimise proteins for maximum fitness. Beginning with a random sequence and a specified ligand, we perform hill-climbing walks on the fitness landscape. The steps made on this landscape are random point mutations of the protein sequence; a mutation is accepted if it results in an increase in the fitness, that is to say, an increase in *P*^0^_rel_ (Bound). This walk is continued until the protein sequence resulting in a local fitness maximum is reached. By calculating the fitness for all single-point-mutants of the sequence, we ensure that the sequence is indeed at a local-maximum. We performed 1000 optimization runs for each ligand. We also perform similar hill-climbing walks designed to maximise *P*(Compact), independent of any fitness based on ligand-binding or catalysis.

## Results

**Figure 2:**
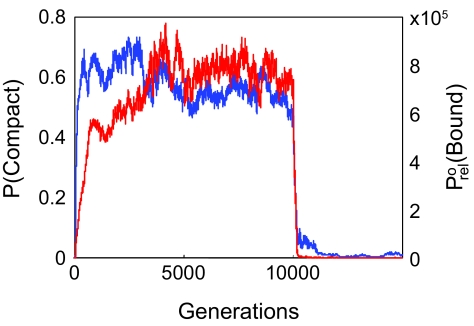
Extended typical evolution run with fitness based on ability to bind and catalyze EEEE, showing the effects of changing *P*^0^_rel_(Bound)_½_. The value of *P*^0^_rel_(Bound)_½_ in the first 10,000 generations equals 15,000, and for the last 5000 generations *P*^0^_rel_(Bound)_½_ equals 1.25. The values plotted are population-weighted averages. Blue line: 〈*P*(Compact)〉, the probability that a protein is in a compact state. Red line: 〈*P*^0^_rel_(Bound)〉, the relative probability that a protein binds a ligand at any site in any conformation. 〈*P*(Native State)〉, the probability of the compact structure with minimum free energy, is indistinguishable from 〈*P*(Compact)〉 throughout the simulation.

The results of a typical evolution run, showing population-weighted averages of *P*(Compact) and *P*^0^_rel_(Bound) are illustrated in Figure 2. The data for the first 10,000 generations are the results of one of the fifty experimental runs with ligand EEEE and *P*^0^_rel_(Bound)_½_ = 15,000. This run has been extended for an additional 5000 generations with a lower *P*^0^_rel_(Bound)_½_ of 1.25. 〈*P*^0^_rel_(Bound)〉 increases steadily and rapidly for several hundred generations, then fluctuates until generation 10,000. This behaviour is due to the semineutral relationship between fitness and *P*^0^_rel_(Bound) described in equation 9. Initially, proteins with higher values of *P*^0^_rel_(Bound) have a selective advantage and are more successful at reproducing, resulting in the increase of 〈*P*^0^_rel_(Bound)〉. As proteins with very high values of *P*^0^_rel_(Bound) emerge and become established in the population, the selective advantage of higher binding probability diminishes, proteins become more equally fit, and the makeup of population becomes more subject to random factors than to fitness effects. After *P*^0^_rel_(Bound)_½_ decreases at generation 10,000, 〈*P*^0^_rel_(Bound)〉 decreases rapidly, not due to selection for weaker binding affinity, but due to the larger number of mutations that decrease rather than increase binding affinity. For both values of *P*^0^_rel_(Bound)_½_, but especially for *P*^0^_rel_(Bound)=15,000 *P*(Compact) is approximately equal to *P*(Native State), indicating that one compact conformation is dominant at equilibrium. It is also generally true that the simulations produce a single dominant binding site, which dominates *P*^0^_rel_(Bound).

**Figure 3:**
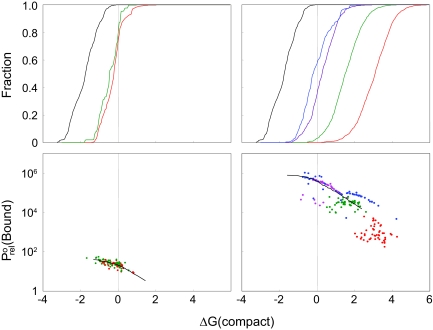
The properties of evolved and optimised proteins, colour-coded by the value of *P*^0^_rel_(Bound)_½_:*P*^0^_rel_(Bound)_½_ = 1.25 (red), 100 (green), 3750 (magenta), and 15,000 (blue). Not all values of *P*^0^_rel_(Bound)_½_ are included in each plot. In the left column (panels **a, b**) the ligand is AAAA, in the right (panels **c, d**), EEEE. **a** and **c**. Cumulative distribution of protein conformational stability. The fraction of final-generation (or optimized) proteins with a AG(Compact) less than the value on the x-axis is plotted on the y-axis. The properties of proteins optimised for compaction are represented in black. **b** and **d**. Scatter 22 plot of relative binding probability vs. protein conformational stability. Each point represents the population average of one simulation at the final generation. Black line represents the averages of approximately 8000 proteins optimised to bind the ligand. Simulations with similar values of Δ*G*(Compact) were binned together into 50 groups, and average values of Δ*G*(Compact) and *P*^0^_rel_ (Bound) computed for proteins in each group.

Figure 3 illustrates the physical properties of the final generations of the evolution experiments for two different ligands, AAAA (3a–3b) and EEEE (3c–3d), compared with the proteins that result from optimization through hill-climbing. Results for different values of *P*^0^_rel_(Bound)_½_ are represented as different colours on the graphs. To examine the stability distribution of the evolved and optimised proteins, we plot cumulative distributions of their levels (calculated with Equation 4) in Figures 3a and 3c. These results are population-weighted, so that common, well-represented proteins influence the distribution more than poorly-represented transients. Most proteins have Δ*G*(Compact) > −1, indicating that proteins are at most marginally stable, consistent with observations of real proteins. This is more clearly evident compared with the distribution of Δ*G*(Compact) values for proteins that have been optimised for stability with a hill-climbing algorithm.

There are several possible explanations for the relationship between overall protein stability and probability of binding a ligand. Thermodynamically, we would expect that a protein with higher stability would, on average, bind more strongly than unstable proteins, due to the entropy penalty when a less-stable protein binds a ligand. Alternatively, the ‘optimization given constraints’ model suggests that there might be a negative correlation between protein stability and binding, as the residues that optimized stability might not be the same as the residues that optimised binding interactions. The positive correlation between binding probability and protein stability shown in Figures 3b and 3d, demonstrates that the thermodynamic effect dominates any possible trade-offs between stability and binding.

Further evidence of the lack of trade-off between ligand binding and protein stability is provided by considering the proteins that have been optimised for binding by the hillclimbing algorithm. We observe no correlation between the stability of the proteins that result and the strength of their binding interactions with the peptide ligand, as would be expected if strong binding interactions were incompatible with high protein stability.

In general, the properties of evolved proteins fall within a range of values, but the ligand and the *P*^0^_rel_ (Bound)_½_ affect the properties of resulting proteins within these bounds. As can be seen in Figure 3, among proteins evolved to bind AAAA there are no discernible changes in the distributions of protein stability, binding probability, and binding interaction strength with different values of *P*^0^_rel_ (Bound)_½_, but among proteins evolved to bind EEEE these quantities tend to increase with *P*^0^_rel_ (Bound)_½_. In addition, the variation in protein properties is higher for proteins evolved with EEEE, especially at lower values of *P*^0^_rel_ (Bound)_½_. Proteins evolved to bind EKEK have similar properties to proteins evolved to bind EEEE.

These differences are due to the nature of the ligand. A mutation can increase *P*^0^_rel_ (Bound)_½_ in two ways: by increasing the complementarity of a binding site or by increasing the probability of a compact state with a favourable binding site. The binding strength of AAAA is of lesser magnitude than that of EEEE, reflecting the difference in the strength of optimal binding interaction. This means that AAAA can only form weak binding interactions; as soon as binding faces evolve that form these interactions, the only way to increase *P*^0^_rel_ (Bound) _½_ is to increase the probability of being in the conformationally correct state. Not all proteins evolved to bind AAAA have formed an optimal-interaction binding site, but binding interactions in most proteins are close to optimal. Proteins evolving to bind EEEE can form stronger binding interactions with a wider variety of binding faces, and a wider variety of successful proteins results. The results for EKEK are similar to those for EEEE. Proteins optimised for ligand binding tend to be relatively stable and have relatively strong binding interactions compared with evolved proteins; in contrast, evolved proteins have higher variation in both Δ*G*(Bound) and Δ*G*(Compact).

## Discussion

Figure 3 shows that most proteins resulting from our evolutionary experiments are not highly stable, consistent with observations about real proteins. In fact, most of the resulting proteins tend to have low or marginal stability levels, depending on ligand and value of *P*^0^_rel_ (Bound)_½_.

As described in the Introduction, there are two different possible reasons generally given for the marginal stability of real proteins - either a specific selective advantage of marginal stability, or a co-optimization of the conflicting qualities of stability and other aspects of functionality. In our model, we have eliminated the first possibility by construction, in that proteins that bind more strongly have a higher fitness. We also observe a positive correlation between stability and fitness, so the theory of conflicting design pressures does not apply in our model. With both of these possible explanations inappropriate for these simulations, we still observe marginally stable proteins.

This provides evidence that sequence entropy, the third possible explanation, is sufficient to explain the observation of marginal stability in biological proteins. We are not ruling out the other proposed explanations, but we can explain the observed properties of evolved proteins without them. The existence of marginal stability in real proteins implies neither an evolutionary advantage to marginal stability nor a trade-off between stability and binding strength. The most parsimonious explanation for marginal stability does not include either of these two mechanisms.

The protein models used in this study are smaller than realistic proteins, and thus might better represent the area surrounding an active site more than an entire protein. Of the three explanations used to explain marginal stability - evolutionary advantage of marginal stability, negative correlation between stability and fitness due to design constraints, and sequence entropy - we would expect the first explanation to be independent of protein size, the second explanation to be especially appropriate around the active site, while the third explanation would involve the entire protein, as the protein generally is required to be folded in order to bind and catalyse a ligand. The fact that we do not observe evidence for the second explanation while the third explanation seems to be adequate in these smaller models suggests that it should also be adequate when a more realistically-sized protein is considered.

The effect of the underlying nature of protein sequence space may have been more important in early protein evolution. Most proteins with random amino-acid sequences are highly unstable (in our model, and likely in the real world), so the first existing proteins would likely have been unstable as well. Some degree of stability was likely beneficial, so mutations that lead to increased stability were accepted. At a certain point, the fitness gains of higher stability were counter-acted by the effect of sequence entropy, and thus protein stability did not increase further.

The resulting stability depends upon the ligand, as well as *P*^0^_rel_ (Bound)_½_. Specific predictions can be made on the basis of this analysis. For instance, we would expect that observed protein stabilities should depend upon the corresponding value of *P*^0^_rel_(Bound)_½_ = exp(−*ΔS*_lig_/*k_B_*)*k_D_*[*L*]/*k*_2_, in that ligands with higher values of *P*^0^_rel_ (Bound)_½_ would likely correspond to more stable proteins. This would be the case for smaller ligands (faster *k_D_*), and slower catalysis (slower *k*_2_). In fact, one might expect that protein stabilities could lessen with time as the catalytic steps became more optimised, reducing the value of *P*^0^_rel_ (Bound)_½_. The current analysis also suggests that highly-sticky proteins (strong binding strength) would correspond to proteins with less stability, as the selective pressure on stability would be reduced. While there are obvious examples of this, such as calmodulin, further investigation is required to see if this is a general principle.

## Appendix

### Appendix: Derivation of the fitness function

To derive the fitness function, we start with equation 8. In the Michaelis-Menten model, the rate of change of [*PL*] is assumed to be zero, so the steady state concentration of *PL* is

(10) $$[PL]=\frac{k_{D}[P][L]}{k_{\text{uni}}+k_{2}}.$$

The total concentration of protein, [*P*]*_T_*, is equal to [*P*]+[*PL*]. Solving for [*P*] and [*PL*] in terms of [*P*]*_T_* yields

(11) $$[P]=\frac{k_{\text{uni}}+k_{2}}{k_{\text{uni}}+k_{2}+k_{D}[L]}{\left[P\right]}_{T}$$

(12) $$[PL]=\frac{k_{\text{D}}[L]}{k_{\text{uni}}+k_{2}+k_{\text{D}}[L]}{\left[P\right]}_{T}.$$

The rate of production of the final product is

(13) $$V=\frac{d[\text{Product}]}{dt}=k_{2}[PL]=\frac{k_{2}k_{D}[L]}{k_{\text{uni}}+k_{2}+k_{D}[L]}{\left[P\right]}_{\tau }.$$

We can relate this to terms calculated from our protein model by expressing *P*^0^(Bound), the relative thermodynamic probability that the protein binds a ligand, in terms of protein and ligand concentrations. *P*^0^(Bound) = [*PL*]/[*P*]*_T_*, under conditions when there is no forward reaction, or *k*_2_=0. Under these conditions, equation 13 becomes,

(14) $$P^{0}(\text{Bound})=\frac{k_{D}[L]}{k_{\text{uni}}+k_{D}[L]}.$$

Solving for *k*_uni_ yields

(15) $$k_{\text{uni}}=\frac{k_{D}[L](1-P^{0}(\text{Bound}))}{P^{0}(\text{Bound})}.$$

Substituting this expression into equation 14 yields

(16) $$V=\frac{k_{D}[L]{\left[P\right]}_{T}}{1+\frac{k_{D}[L]}{k_{2}P^{0}(\text{Bound})}}.$$

We assume that *k**_D_*, [*P*]*_T_*, and [*L*] remain relatively constant, and as fitness is relative and thus not changed by a multiplicative factor, the final fitness function is then:

(17) $$\text{Fitness}=\frac{1}{1+\frac{P^{0}{\left(\text{Bound}\right)}_{1/2}}{P^{0}(\text{Bound})}}$$

where *P*^0^(Bound)_½_ = *k**_D_*[*L*]/*k*_2_ is the value of *P*^0^(Bound) where the fitness is half the maximum fitness.
